# Unlocking the potential of fungi: the QuantFung project

**DOI:** 10.1186/s40694-015-0016-0

**Published:** 2015-09-16

**Authors:** Zsófia Büttel, Rafael Díaz, Benedict Dirnberger, Michal Flak, Sietske Grijseels, Min Jin Kwon, Jens Christian Froslev Nielsen, Yvonne Nygård, Pradeep Phule, Carsten Pohl, Sylvain Prigent, Milica Randelovic, Tabea Schütze, Danielle Troppens, Annarita Viggiano

**Affiliations:** 1 Department of Molecular Microbiology, Groningen Biomolecular Sciences and Biotechnology Institute, University of Groningen, Groningen, The Netherlands; 2 Department of Biochemical Engineering, Faculty of Sciences and Technology, University of Debrecen, Debrecen, Hungary; 3 Department of Molecular Microbiology and Genetics, Georg-August-University Göttingen, Groningen, Germany; 4 Department of Molecular and Applied Microbiology, Leibniz Institute for Natural Product Research and Infection Biology, Hans Knöll Institute, Jena, Germany; 5 Department of Systems Biology, Technical University of Denmark, Kongens Lyngby, Denmark; 6 Department of Applied and Molecular Microbiology, Institute of Biotechnology, Applied and Molecular Microbiology, Berlin University of Technology, Gustav-Meyer-Allee 25, 13355 Berlin, Germany; 7 Department of Chemical and Biological Engineering, Chalmers University of Technology, Gothenburg, Sweden; 8 Department of Genetics and Molecular Biology in Botany, Institute of Botany, Christians-Albrechts-University, Kiel, Germany

## Background

The crisis of antibiotic resistance has been much discussed in recent decades within research communities and also in public. However, options to slow down the spread of resistance and the opportunities to discover new antimicrobial agents seem to be limited at present. Can we, by unlocking the hidden potential of fungi, attain new means to gain advantage in this battle?

Since the discovery and global use of the first fungal-derived antibiotic, penicillin, resistance emerged. As new and larger quantities of antibiotics were introduced to the market, the more rapidly resistance was observed. This development was fostered by substantial over-use and misuse of antimicrobial substances in the treatment of human diseases, as well as their concurrent introduction in agriculture and veterinary settings. In addition to the prominent examples of bacteria, antimicrobial resistance is also advancing in causal agents of notorious infectious diseases such as malaria (a parasite), influenza and HIV (viruses), candidiasis and candidaemia (fungi), all of them posing a major threat to the public health, that collectively cause hundreds of thousands deaths every year.

Especially in recent years, national and international programmes and campaigns have been initiated to raise the public awareness on antibiotic resistance. The World Health Organization is monitoring the use of antibiotics and the emergence of resistant microbes, especially in countries where surveillance is virtually non-existent. Likewise, in Europe, US or Australia, where antibiotic usage is already more restricted as a response to concerns about drug resistance, public campaigns such as the “European Antibiotic Awareness Day”, “Get Smart about Antibiotics” or “National Antimicrobial Resistance Strategy” respectively urge for a more sensible and careful use of antibiotics.

In addition to raising public awareness, the US and Europe are joining forces to encourage and renew efforts into research for novel antimicrobial substances and targets. This has been hampered by the decreasing interest of the pharmaceutical sector to invest in the development and testing of drugs that are perceived as unprofitable by virtue of a limited financial market and other costs such as to be ineffective incentives. The problem was summarized in a comment in *Nature* by Cooper and Shlaes [1] (“Fix the antibiotics pipeline”) and is described in more detail in Shlaes’s book “Antibiotics: The Perfect Storm” [2], illustrating the requirement for new approaches to tackle microbial diseases by pointing out the steady decline in the number of new antimicrobial drugs entering the market and the dramatic increase of resistant bacteria. One of the EU/US joined task forces, the Transatlantic Task Force on Antimicrobial Resistance (TATFAR) that started in 2009, aims to facilitate financial benefits for research and improve the pipeline for the market approval of new drugs.

Around the time of these initiatives, at a conference in 2011 on synthetic biology, held in St. Feliu in Spain, scientists devoted to fungal research discussed the great potential of fungi to produce natural products and how little we still knew about those products. It had not been long before that the genomes of two biotechnologically relevant fungi, *Penicillium chrysogenum* (production of penicillin) [3] and *Aspergillus niger* (production of citric acid) [4], had been published and gene clusters predicted to be involved in the synthesis of known and unknown secondary metabolites identified. It was clear that these fungi could potentially produce many more substances than those we were already using. Thus, there was a demand for new manners to discover and induce production of fungal metabolites. Therefore, a project proposal was written to intensify the efforts to find novel antimicrobial fungal products while also to provide the training framework to educate the future generation of young researchers.

The project was provided with €3.9 million (EU Grant number 607332) as a 4-year multi-partner initial training network (ITN), as part of the Marie-Curie Actions in the Seventh Framework Programme ‘People’ (FP7 people), and it started in October 2013. Its focus is “Quantitative Biology for Fungal Secondary Metabolite Producers”, or in short “QuantFung”. It involves seven universities and a research institute, one industrial company as a full partner and three associated industry partners, and is located in five different countries. We, the fellows, were recruited as young researchers, eleven of us as early stage researchers (ESR) aiming for a PhD, and four of us as experienced researchers (ER) in postdoctoral positions. In the coming years, we envision to make a contribution to new methods for finding and producing novel bioactive molecules from fungi.

## The fellows behind QuantFung

**Fig. 1:**
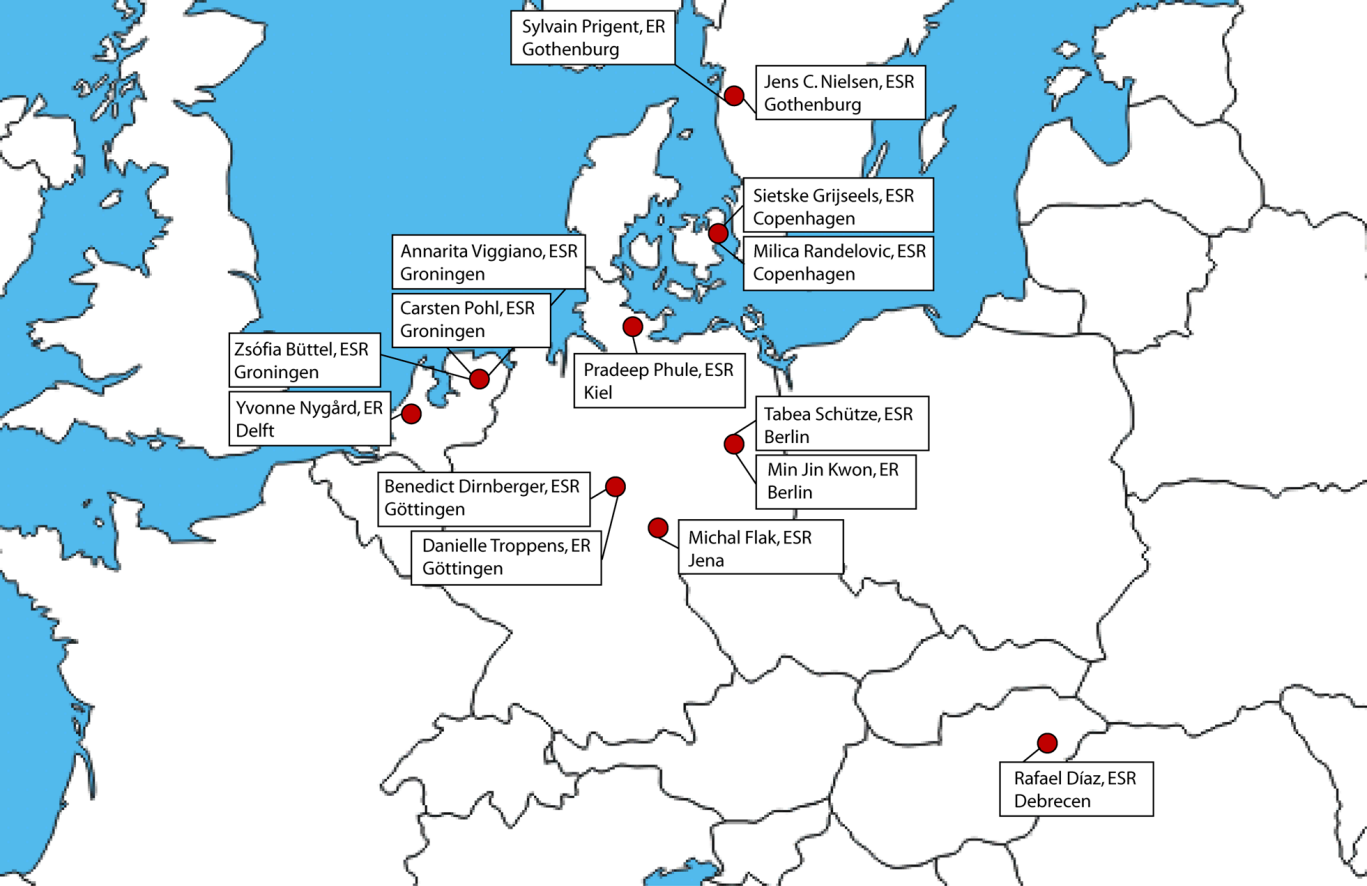
Locations of the fellows. This map lists the early stage researchers (ESR) and experienced researchers (ER) of our initial training network and the city where they are located

In order to be accepted for a position within an EU-funded ITN, each fellow needed to fulfil eligibility criteria. One condition was to move to a country not lived in during the past 3 years. Given this mobility rule, we fellows come from 12 different nations and are working in 5 different countries (Fig. 1). We all have different backgrounds, scientifically, as well as culturally, allowing us to improve our scientific and networking skills. The ITN is an excellent way of training our communication skills as well as finding common ground for collaborations.

In addition to equipping us with the skills to fulfil our tasks within the ITN, the network provides a wide range of training possibilities and serves as a bridge between industry and academia. The consortium organizes workshops in different disciplines: In the first year of QuantFung, for instance, most of us joined a summer school on Quantitative Biology. Other courses, e.g. on proteomics or entrepreneurship, are yet to come. Another important part of our ITN is the so called “secondments”. A secondment can last for 1-6 months and should address one part of our research that can be done in the facilities of a partner. The idea behind these secondments is to strengthen collaboration and to experience the environments and laboratory practices in another lab and institutions. Selecting an industrial partner for a secondment provides the experience of a different attitude towards research and to consider important aspects of research such as sustainability or time demand for our research questions.

Besides the numerous positive aspects of a Europe-wide project we are also aware of the challenge of staying connected between multiple locations and keeping up to date with the research endeavours of others. With this in mind, the consortium meets twice a year. By presenting our research at these meetings we stimulate knowledge transfer and also ensure staying focused with valuable critique and fruitful discussions by sharing and combining ideas and strategies. This is an essential part of scientific work that we have the opportunity to “practice” within a training environment.

## Divide and conquer

**Fig. 2:**
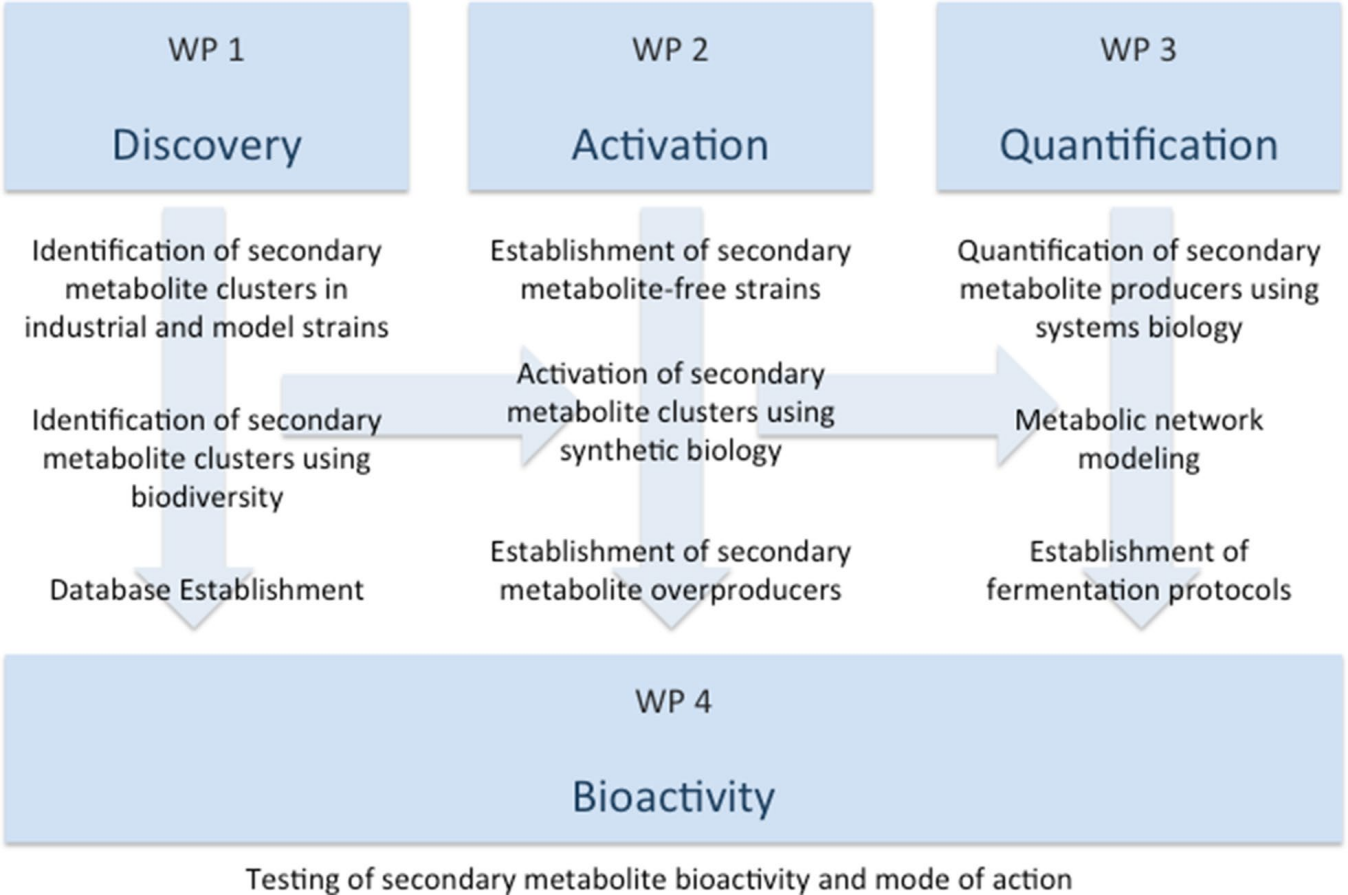
Workflow overview of QuantFung. All work packages can start individually but cooperate and share results in later stages of the project

Our tasks are divided into four work packages, as outlined in Fig. 2. Each work package has an independent starting point to ensure that each fellow can start their research at the time of recruitment. In work package one, our aim is to identify novel gene clusters that produce secondary metabolites in several fungal species. By using sequencing technology, we will obtain an inventory of genes potentially useful for secondary metabolite production. An interesting feature of genes involved in formation of a secondary metabolite is their clustered arrangement in the genome. This makes the identification of the genes responsible for metabolite biosynthesis, by way of the physical proximity of the genes possible, based on searching for key genes such as the non-ribosomal-peptide synthetases or the polyketide synthases.

Besides industrial fungal strains with sequenced genomes such as *Aspergillus* spp. or *Penicillium* spp., novel fungal species will be isolated, sequenced and potentially explored. One of us (Pradeep Phule) is sequencing fungi isolated from marine and terrestrial habitats. These uncharacterised species are being investigated by sequencing their genomes and using computational tools to identify conserved features in genes which are involved in secondary metabolite production.

The gene clusters found with this bioinformatics approach will then be expressed in heterologous host fungal species in work package two. This is expected to lead to a number of novel products, the bioactivities of which are to be determined in work package four. In addition to the sequence data gathered specifically by QuantFung, the emerging genome sequences of fungal strains will be explored. For instance, the program will tap into the resources of the major effort within this field, being the sequencing of 1000 previously unsequenced fungi by the Joint Genome Institute (Department of Energy, USA).

The main experiments of work package two are the expression of secondary metabolite genes which were discovered from genome mining. Genes within secondary metabolite clusters will be transferred into *Aspergillus niger* (Tabea Schütze), *Aspergillus nidulans* (Benedict Dirnberger, Danielle Troppens) or *Penicillium chrysogenum* (Carsten Pohl, Annarita Viggiano) under the control of different promoters to drive their expression. In addition, work package two includes the production of strains that can serve as chassis or platform strains. Targeted gene replacement or the CRISPR/Cas9 system will be used to mutate the genes for the endogenous secondary metabolites in the host species. A secondary metabolite-free strain would allow easier downstream detection and purification of the secondary metabolite of interest. To boost the expression of genes that are normally silenced, we will also use synthetic biology tools that enhance transcriptional activation such as the tetracycline inducible tet-system or similar inducible tools. The development of modular gene expression systems (Carsten Pohl, Tabea Schütze, Yvonne Nygård, Zsófia Büttel, Annarita Viggiano) and the study of a new platform to produce bioactive substances in specialized fungal cells (Benedict Dirnberger) are two other exciting projects within our ITN where we anticipate contributing new tools and strategies to the fungal community.

Gene expression and metabolite production need to be as efficient as possible. Therefore, work package three is focused on systems biology and optimisation of cultivation conditions for secondary metabolite producers. We will generate metabolome and transcriptome data under controlled conditions, e.g. using chemostat and batch cultivations. By incorporating these data into existing metabolic models for *Penicillium* spp. (Jens Christian Nielsen, Milica Randelovic, Carsten Pohl, Sietske Grijseels, Min Jin Kwon), we want to improve the understanding of fluxes within the fungal cell towards secondary metabolites and identify key reaction steps to focus on further flux modelling and optimisation (Sylvain Prigent). Because our project could potentially identify interesting compounds that would require synthesis in larger quantities for bioactivity screenings or structure elucidations, work package three will also produce cultivation protocols for improved secondary metabolite production in lab and pilot scale bioreactors (Rafael Diaz, Milica Randelovic).

Looking at the three previously described work packages, a pipeline of steps will ideally lead to the production of novel secondary metabolites. However, fast screenings of those metabolites for various applications are a prerequisite to decide if a certain molecule is of relevance for further studies. Thus, work package four addresses the bioactivity of secondary metabolites. In work package four, Annarita Viggiano and Michal Flak will analyse the activity of novel secondary metabolites against multi-drug resistant bacteria (e.g. *Pseudomonas aeruginosa, Staphylococcus aureus, Klebsiella pneumoniae*) and other relevant microorganisms (e.g. fungal pathogens like *Candida* spp.). Together with our industrial partners, we will also evaluate the potential for other applications ranging from antifungal activity for crop protection to applications in medicine beyond anti-infective treatments.

## Quo Vadis, QuantFung?

**Fig. 3:**
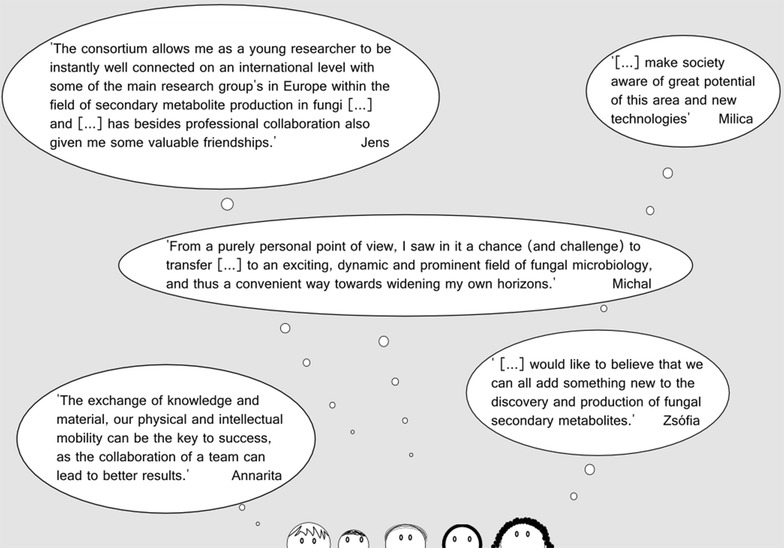
Opinions on the impact of QuantFung. This shows some thoughts on our motivation and why we believe that QuantFung will be a successful project

What is it that we want to achieve within the next years? What should we be aware of for our future careers? What contribution do we wish to make with this ITN to society? In the following, we share some of our thoughts and our ideas on how we see this ITN impacting the fungal community and our careers (see also Fig. 3).

We believe that QuantFung could make a substantial contribution to new tools and methods for research with filamentous fungi. With our project, we can develop new tools and also adapt techniques to fungi from other organisms, where synthetic biology tools, metabolic modelling and clever strategies for gene expression are already established. We would like to stress that the tools we develop and the knowledge we generate could also be utilised for applications beyond secondary metabolite discovery. In our opinion, the potential of filamentous fungi for biotechnological production processes is not yet fully exploited, perhaps also due to the lack of simple, efficient methods and protocols for genetically modifying these organisms and to perform straightforward, metabolic engineering.

The public has, in recent years, especially in the EU, shown enormous resistance towards growing genetically modified organisms (in particular crops) for food and feed purposes. This, however, seems not to be the case for engineered organisms that produce life-saving drugs such as insulin, other hormones, antibiotics, cholesterol-reducing agents like lovastatin, or the immunosuppressant cyclosporins that underlies successful organ transplantation. Even though this might be the result of these types of molecules not being publically discussed at an equal extend as GMOs as food sources, we should take the scepticism about genetic modifications amidst our audience seriously.

Especially when working with unknown isolates and strains capable of producing potentially harmful substances, in the communication with the public we will clarify that we are aware of the measures to be taken to prevent any harm to humans, other species, and the environment. In brief: we are ambassadors of good laboratory practice and we regard it as our task to communicate that synthetic biology and genetic engineering is sound. We should have a concrete idea about the work we do and be able to explain how society will benefit from our research. Consequently, we would like to contribute to improving the acceptance of new, yet safe scientific methods.

Last but not least, when finishing this project in 2 or 3 years from now we aim to have gained a problem-solving attitude that will help us adapt to new challenges and quickly recover from drawbacks. We should also keep in mind that establishing and maintaining a vital network for exchanging ideas about our research is most fruitful if it actually leads to collaborations. So, it will require our continuous initiative to develop our stories as part of the ITN and to intertwine them. In that sense, we envision QuantFung as a catalyst or a platform to ease this kind of cooperation. Ideas, visions, and true need for cooperation are essential to bring people together. To extend this, we are interested in connecting with other current ongoing fungal ITNs, namely YeastCell, FungiBrain and ImResFun. Together with fellows of YeastCell and FungiBrain, we already held a successful symposium in Marburg (Germany) during the Annual Meeting of the Association for General and Applied Microbiology (VAAM). In addition, during our first retreat, a gathering of all QuantFung ESRs and ERs in Prague (Czech Republic), we reached out to master students at the Charles University and presented our ITN and the Marie-Curie Actions programme. At this occasion, we extended our network again by inviting a fellow of ImResFun to introduce his ITN to us and the students.

Thus, the foundations have been laid and the future will show how we and this type of initiative will succeed in establishing collaborations and a network with fellow research groups with expertise in different methods and topics. With this in mind, we are convinced that QuantFung is an excellent opportunity to start a research-oriented career in natural sciences.
